# Strengthening ties towards a highly-connected world

**DOI:** 10.1007/s10618-021-00812-1

**Published:** 2022-01-04

**Authors:** Antonis Matakos, Aristides Gionis

**Affiliations:** 1 Aalto University, Espoo, Finland; 2 KTH Royal Institute of Technology, Stockholm, Sweden

**Keywords:** Strong triadic closure, $\textsc {stc}$, Link recommendations, Densest subgraph discovery

## Abstract

Online social networks provide a forum where people make new connections, learn more about the world, get exposed to different points of view, and access information that were previously inaccessible. It is natural to assume that content-delivery algorithms in social networks should not only aim to maximize user engagement but also to offer opportunities for increasing connectivity and enabling social networks to achieve their full potential. Our motivation and aim is to develop methods that foster the creation of new connections, and subsequently, improve the flow of information in the network. To achieve our goal, we propose to leverage the *strong triadic closure* principle, and consider violations to this principle as opportunities for creating more social links. We formalize this idea as an algorithmic problem related to the densest *k*-subgraph problem. For this new problem, we establish hardness results and propose approximation algorithms. We identify two special cases of the problem that admit a constant-factor approximation. Finally, we experimentally evaluate our proposed algorithm on real-world social networks, and we additionally evaluate some simpler but more scalable algorithms.

## Introduction

In the past decade we have witnessed social networks becoming an integral part of society. Social networks like Facebook, Twitter and LinkedIn have grown steadily in recent years, attracting billions of users, and becoming a staple in our everyday life. Users of these networks are offered new ways of interacting with each other, while discovering new people and creating friendships; people nowadays tend to have hundreds of online connections (Ugander et al. 2011). In reality, however, since meaningful interactions require time and effort, not all connections in a network correspond to strong friendships; in fact, most connections correspond to acquaintances.

The distinction between close friends and acquaintances is an important dichotomy we need to make when studying the dynamic behavior of friendships in a social network. Understanding these dynamics is key for the study of many fundamental network concepts. The strength of ties plays a critical role in how information flows in the network, how people get acquainted with each other, and how the structure of the network evolves over time.

An attractive principle from sociology, which can help us understand the dynamics of the strength of social connections on social networks is the *strong triadic closure* ($\textsc {stc}$) principle. In simple terms, $\textsc {stc}$ states that if two people in a social network have a close friend in common, then there is an increased likelihood that they will become acquainted at some point in the future (Rapoport 1953). More formally, given a classification of social ties into *strong* and *weak*, $\textsc {stc}$, in its most rigid form, states that if an individual *A* has strong ties to individuals *B* and *C*, then *B* and *C* need to have a tie (either strong or weak) between themselves. Strong triadic closure is an intuitive notion having grounds in sociology (Catton 1962). Furthermore, the experiments of Granovetter (1973) and later Easley and Kleinberg (2010) provided empirical evidence for the validity of $\textsc {stc}$ in real-world social networks.

Recent work on using $\textsc {stc}$ for social-network analysis has mainly focused on inferring the strength of social ties. In one of the first works, Sintos and Tsaparas (2014) search for an assignment of tie strengths, which maximizes the number of strong edges, while ensuring that the $\textsc {stc}$ property is respected over the whole network. Subsequent works refined this methodology by studying less rigid versions of the $\textsc {stc}$ property (Adriaens et al. 2020), as well as considering the interplay with community structure (Rozenshtein et al. 2017).

While these works have initiated the study of $\textsc {stc}$ around algorithmic problems, they use the $\textsc {stc}$ property to infer the strength of ties in a *snapshot* of the network. From this perspective, our work is a departure from the previous ways of thinking about $\textsc {stc}$. Instead of using $\textsc {stc}$ to characterize a static network, we assume that $\textsc {stc}$ describes a *mechanism* by which new connections are formed. Therefore, by assuming that we already know the tie strength, we propose to leverage this mechanism by making content recommendations that will strengthen some ties and, according to $\textsc {stc}$, lead to the formation of new ties. The goal is to select the connections that, according to the $\textsc {stc}$ property, increase the potential of new social connections.

**Fig. 1:**
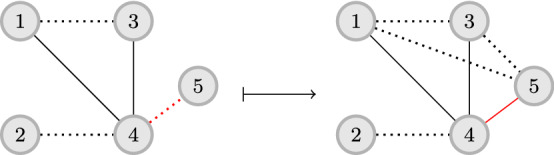
Illustration of the effect of the $\textsc {stc}$ principle. Solid edges correspond to strong ties and dashed edges to weak ties. Observe (in red) the effect of strengthening tie (4, 5): there is increased chance for ties (1, 5) and (3, 5) to be created. On the other hand, the $\textsc {stc}$ principle does not stipulate creation of tie (2, 5) as (2, 4) is weak (Color figure online)

Our guiding principles are the following. First, fostering new network connections ensures people have more opportunities to meet and create new friendships, thus, maximizing user engagement. In addition, higher connectivity improves the flow of information in the network. Second, we want to achieve our objective with as little external intervention as possible. By using $\textsc {stc}$ we can organically create new links, by only reinforcing existing links. Finally, as we will demonstrate more clearly further, an edge-strengthening recommendation could have a higher impact on the objective, since a single strengthening could result in the formation of many new edges.

Our problem formulation is centered around the assumption that according to the $\textsc {stc}$ principle, two people with a common close friend have a higher opportunity to meet and form a new connection. We refer to these connections as $\textsc {stc}$ *bridges*. We also assume that the social network may present opportunities for two people to get to know each other better and strengthen their friendship. Putting together the above ideas, we aim to maximize the number of $\textsc {stc}$ bridges in the network by turning some edges from weak to strong. Note that a single edge might be part of multiple $\textsc {stc}$ bridges, maximizing the potential of strengthening that edge. The example in Fig. 1 demonstrates the effect of an edge strengthening.

We assume that tie strengthening can be achieved in the form of a feature offered by the social network to users who want to opt into. Such a feature would prioritize content from certain users who form weak ties with the user, with the objective of strengthening the tie. It is worth noting that Facebook has experimented with similar ideas, as reported in the media.^1^ One can also leverage existing works for strengthening ties in the context of $\textsc {stc}$ (Gilbert and Karahalios 2009; Torro and Pirkkalainen 2017).

We note that our approach is graph-driven instead of user-driven. In particular, we aim to utilize the structure of the social graph, instead of data on user behavior. We believe this ensures that the privacy of the users is better respected, while requiring a minimal amount of data. Additionally this approach leads to an interesting problem formulation, which in turns allows us to develop novel algorithmic ideas. Resulting from this line of thinking we make some modeling assumptions. First, we assume that the question of how to strengthen a tie is specific to a social network, while we aim at an abstract problem formulation that can form a basis for strengthening ties in several different social networks. Therefore, we ask the question: “Given a content recommendation mechanism that has the capability to bring two acquainted people closer together, which ties should we strengthen, in order to maximize the resulting number of connections?” Additionally, in practice it is not equally easy to strengthen each tie. However, since the only distinction we make under $\textsc {stc}$ is into strong and weak ties, we assume that all weak ties can be converted to strong with equal difficulty. Naturally, the trade-off of such an assumption is that it may lead to some recommendations that are uninteresting to the user, given that our approach does not account for user preferences. Additional limitations of our approach and the impact of our assumptions are discussed in more detail in Sect. 10.

Another consideration is that we want to minimize the disruption of the organic structure of the network. To accomplish this objective we consider a limit on how many ties can be converted from weak to strong, by introducing a budget *k*.

On a more technical level, our problem formulation presents an interesting mapping to a variant of the densest *k*-subgraph ($\textsc {d}k\text {s}$) problem, which is at the crux of our algorithmic results. From an empirical perspective, we experimentally evaluate our proposed algorithm, in addition to evaluating some simpler but more scalable algorithms.

In summary, we make the following contributions:

- We leverage the strong triadic closure ($\textsc {stc}$) property in a novel way, for the task of maximally increasing the connections in a social network. We formulate the task as a formal algorithmic problem, which we call MaximizeSTCBridges.
- We prove that the MaximizeSTCBridges problem is $\mathbf {NP}$-hard and give approximability results.
- We study the algorithmic properties of our problem in connection to a novel variant of the $\textsc {d}k\text {s}$ problem.
- We identify special cases of the problem for which a constant approximation factor can be guaranteed.
- In the experimental section, we propose strong baselines and compare the performance of our algorithm against these baselines.

The rest of the paper is organized as follows. We first put our work in perspective and discuss related work in Sect. 2. Then we present our problem formulation in Sect. 3, while the problem complexity is studied in Sect. 4. In Sect. 5 we reveal the connection of the problem we formulate in this paper with the densest-subgraph problem, and in Sect. 6 we present our algorithm. In Sect. 7 we study properties of the wedge graph, which is used in our construction, and based on these properties, in Sect. 8 we identify two problem variants that admit constant-factor approximation guarantees. In Sect. 9, we present our experimental evaluation for the proposed methods. Finally, in Sect. 10 we discuss limitations of our approach, while Sect. 11 offers a short conclusion and directions for future work.

## Related work

This paper focuses on leveraging the *strong triadic closure* ($\textsc {stc}$) property for a novel algorithmic problem. The concept of strong triadic closure was first introduced by Simmel (1908), but it was made popular by Granovetter in his 1973 paper *“the strength of weak ties”* (Granovetter 1973). More recently, the concept was brought again to the forefront in the book of Easley and Kleinberg *“Networks, Crowds and Markets: reasoning about a highly connected world”* (2010), who posit that strong triadic closure occurs in a social network because there is increased opportunity for vertices with a common neighbor to meet, and therefore, create at least weak ties.

Sintos and Tsaparas (2014) study the problem of labeling the edges of the graph to maximize the number of strong edges, such that the assignment satisfies the $\textsc {stc}$ property. Subsequently, Rozenshtein et al. (2017) consider the problem of the inference of social tie strength, while also taking community structure into account. A recent work by Adriaens et al. (2020) builds directly on the $\textsc {stc}$-inference problem posed by Sintos and Tsaparas by extending and relaxing their formulation via introducing new constraints and integer labels. While these works use the $\textsc {stc}$ property in order to characterize the ties currently present in the network, we view the $\textsc {stc}$ property as a *process* that takes place in the network and leads to the creation of new edges.

Our paper also shares similarities with other lines of work that consider the introduction of new edges in a social network to improve specific properties. Parotsidis et al. consider adding edges to increase user centrality (Parotsidis et al. 2016), while other works have focused on improving shortest path distance (Meyerson and Tagiku 2009; Papagelis et al. 2011; Parotsidis et al. 2015), diameter (Demaine and Zadimoghaddam 2010), eccentricity (Perumal et al. 2013), communicability (Arrigo and Benzi 2015), and connectivity (Chan et al. 2014). Since in Sect. 8 we consider a problem variant that aims to strengthen so called “local bridges,” our work is also similar to the approach of Garimella et al. (2017), who consider the problem of creating bridges to connect communities with opposing views. To the best of our knowledge, this is the first work to take advantage of the $\textsc {stc}$ property for the task of increasing network connectivity.

Central to our work is the well-studied *densest k*-*subgraph* ($\textsc {d}k\text {s}$) problem. Given a graph *G* and a parameter *k*, the $\textsc {d}k\text {s}$ problem asks to find a subgraph of *G* on *k* vertices with maximum density. The $\textsc {d}k\text {s}$ problem has been shown to be $\mathbf {NP}$-hard and it does not admit a $\textsc {ptas}$ under the assumption that $\mathbf {NP}$ does not contain sub-exponential time algorithms (Khot 2004). The work of Chen et al. (2010), which focuses on the $\textsc {d}k\text {s}$ problem on several classes of intersection graphs, provides some essential results for our paper. In particular, our approach relies on adapting their algorithm for a novel variant of $\textsc {d}k\text {s}$, the $k{{\text {-}}\textsc {Densify}}$ problem.

Drawing further inspiration from the work of Chen et al. we adopt the notion of $\sigma$-quasi elimination orders, which generalize perfect elimination orders for chordal graphs. The notion of a $\sigma$-quasi elimination order was first proposed by Akcoglu et al. (2002). Ye and Borodin (2009) investigated further the properties of $\sigma$-quasi-elimination orders for various graph classes and initiated the study of their algorithmic aspects. Finally, Chen et al. (2010) propose a $\mathcal {O} (\sigma )$-approximation algorithm for the $\textsc {d}k\text {s}$ problem if the graph has a polynomial time computable $\sigma$-elimination order. In our work we study the $\sigma$-quasi elimination properties of a special type of graph that is of interest, and use the properties to derive constant-factor approximation guarantees, in some special cases.

## Problem formulation

Let $$G =(V,E)$$ be an undirected graph that represents a social network. The set of vertices $$V$$ represents individual users, and the set of edges $$E$$ represents social connections between the individual users. When refering to a subset of vertices $$X\subseteq V $$ and all edges between them, we will refer to the *induced* subgraph of $$G $$, and denote it as $$G [X]$$.

We consider a labeling $$\ell $$ on the edges of the graph, indicating whether each edge $$\{v,w\}$$ in $$E $$ corresponds to a *strong* ($$\texttt {S} $$) or *weak* ($$\texttt {W} $$) social connection. In particular, this edge labeling is represented as a function $$\ell : E \rightarrow \{\texttt {W},\texttt {S} \}$$. A pair of *incident* edges $$e_1=\{u,v\}\in E $$ and $$e_2=\{u,w\}\in E $$ where $$\{v,w\} \notin E$$ is called a *wedge*. We write $$({e_1}\wedge {e_2}) $$ to denote the wedge between edges $$e_1$$ and $$e_2$$. The set of all the wedges in the graph is denoted by $$W$$.

The *strong triadic closure* ($\textsc {stc}$) property states that if a vertex *v* has strong ties to vertices *u* and *w*, i.e., if $\ell (\{v,u\})=\texttt {S}$ and $\ell (\{v,w\})=\texttt {S}$, then *u* and *w* are more likely to form an edge in $E$, which can be either a weak or a strong tie (Easley and Kleinberg 2010). The absence of the edge $\{u,w\}$, in the presence of strong ties for $\{v,u\}$ and $\{v,w\}$ is called an $\textsc {stc}$ *violation* (Sintos and Tsaparas 2014).

### Definition 1

($\textsc {stc}$ violation) Given a graph $G =(V,E)$ and a labeling function $\ell$ from the edges of $G$ to $\{\texttt {W},\texttt {S} \}$, a triple of vertices $v,u,w \in V$ constitutes an $\textsc {stc}$ *violation* if $\ell (\{v,u\})=\texttt {S}$, and $\ell (\{v,w\})=\texttt {S}$, and $\{u,w\} \notin E$. We will denote as $\mathcal {B} (\ell ,G)$ the total number of violations on the graph $G$ induced by the labeling $\ell$.

The strong triadic closure suggests a structural property that is likely to be true among triples of vertices, but obviously one should not expect it to always hold. A given graph $G$ with a given labeling $\ell$ may have a large number of violations. In this paper we consider an $\textsc {stc}$ violation as an event that may lead to the formation of new social connections in the graph: two edges $\{v,u\}$ and $\{v,w\}$ with strong ties suggest the possibility for *u* and *w* to get acquainted and form a connection. Thus, we view an $\textsc {stc}$ violation as an opportunity for a spontaneous social connection. For this reason we will say that an $\textsc {stc}$ violation leads to an $\textsc {stc}$  *bridge*.

Our goal is to maximize the number of social connections in the network. Since we assume $\textsc {stc}$ bridges will lead to the formation of new edges, we aim to maximize the number of $\textsc {stc}$ bridges. Notice that the network may already contain $\textsc {stc}$ bridges, which however have not yet materialized into new weak edges.

In order to maximize the number of $\textsc {stc}$ bridges we will be looking to convert some edges from weak to strong. We assume this can be achieved through content recommendations for users who opt-in to a feature provided by the social network. An example of such functionality would be to prioritize content generated by a connection in the user’s timeline. Such a functionality could present more opportunities for the users to interact with each other. However, as we mentioned, in this paper we focus on the question of *which* ties to select to strengthen, and we consider the problem of *how* to strengthen them, to be orthogonal to our problem. Therefore, for the sake of concreteness and ease of presentation, we consider a simplified setting where each user opts in to receive content recommendations from other users, and also that it is equally difficult to convert each tie, without considering user preferences. Finally, we consider that heavy interference with the natural structure of the network may harm user experience. To amend this, we consider a limit on how many ties can be converted from weak to strong, by introducing a budget *k*.

Consider two edge labelings $\ell$ and $\ell ^{\prime }$ on the graph $G$. We say that $\ell ^{\prime }$ is a *k*-*strengthening* of $\ell$ if there is a set $E ^{\prime } \subseteq E$ of *k* edges (i.e., $|E ^{\prime } |= k$) such that (*i*) for each $\{u,v\}\in E ^{\prime }$ it holds that $\ell (\{u,v\})=\texttt {W}$ and $\ell ^{\prime } (\{u,v\})=\texttt {S}$, and (*ii*) for each $\{u,v\}\in E \setminus E ^{\prime }$ it holds that $\ell (\{u,v\})=\ell ^{\prime } (\{u,v\})$.

Considering the previous discussion we now formulate the problem that we study in this paper.

### Problem 1

(MaximizeSTCBridges) Given a graph $G =(V,E)$ and a labeling function $\ell$ from the edges of $G$ to $\{\texttt {W},\texttt {S} \}$, find a labeling $\ell ^{\prime }$ that is a *k*-strengthening of $\ell$ and the number of $\textsc {stc}$ bridges ${\mathcal {B}} (\ell ^{\prime },G)$ induced by $\ell ^{\prime }$ on $G$ is maximized.

## Problem complexity

In this section we establish the complexity of Problem 1. We first define the notion of density and formally introduce the densest *k*-subgraph ($\textsc {d}k\text {s}$) problem.

### Definition 2

(Density) Consider an undirected graph $G =(V,E)$. The density of a non-empty subset of vertices $X\subseteq V$ is defined by $\rho (X)=\frac{|E(X)|}{|X|}$, where *E*(*X*) is the set of edges in the induced subgraph $G [X]$.

### Problem 2

($\textsc {d}k\text {s}$) Given an undirected graph $G=(V,E)$ and an integer $k$, the $\textsc {d}k\text {s}$ problem asks to find a subset of vertices $S \subseteq V$ such that $|S | = k$ and $\rho (S)\ge c$ (decision version).

We are now ready to show a reduction from the $\textsc {d}k\text {s}$ problem to our problem. We will consider a decision variant of MaximizeSTCBridges, where we ask for a *k*-strengthening of the labeling function $\ell ^{\prime }$ such that ${\mathcal {B}} (\ell ^{\prime },G)\ge c$. We call this variant MaximizeSTCBridges-*d*. The decision variant can be easily converted into the optimization variant.

### Lemma 1

The problem MaximizeSTCBridges-*d* is $\mathbf {NP}$-complete.

**Fig. 2:**
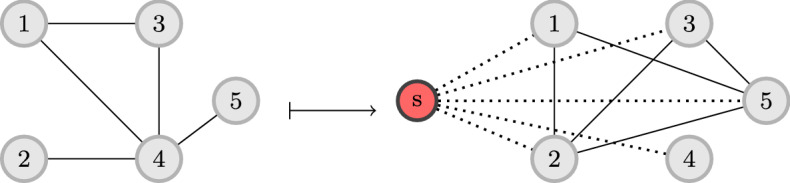
Construction of graph *H* used in the proof of Lemma 1

### Proof

Given a graph $G=(V,E)$ input to the $\textsc {d}k\text {s}$ problem, we create an instance of the MaximizeSTCBridges-*d* problem as follows: we consider the complement of *G*, which we denote by ${\overline{G}}=(V,{\overline{E}})$, and we define by $\{u,v\}\in {\overline{E}}$ if and only if $\{u,v\}\not \in E$. We consider an additional vertex *s*, which is connected to all other vertices in *V*. We denote by $E_s$ the set of edges that are incident to *s*, i.e., $E_s=\{\{s,v\}\mid v\in V\}$. We then construct a new graph $H=(\{s\}\cup V, E_s \cup {\overline{E}})$. Additionally, we introduce a labeling $\ell$ so that $\ell (e)=\texttt {W}$ for all $e\in E_s$ and $\ell (e)=\texttt {S}$ for all $e\in {\overline{E}}$. It is straightforward to see that the graph *H* can be constructed in polynomial time. An example for the construction of graph *H* can be seen in Fig. 2.

We ask for a solution $\ell '$ of MaximizeSTCBridges-*d* on the graph *H*, such that ${\mathcal {B}} (\ell ^{\prime },H)\ge c$. The first key observation is that no edge $e \in {\overline{E}}$ can be in the set of strengthened edges in the *k*-strengthening returned as the solution to the MaximizeSTCBridges-*d*, since it is already $\ell (e)=\texttt {S}$. Additionally, we observe that there is no added benefit from any combination of $e_1, e_2$ with $e_1 \in E_s$, $e_2 \in {\overline{E}}$, and $\ell '(e_1)=\ell '(e_2)=\texttt {S}$. This follows from the construction of *H*, since for any $e_1=\{s,u\}\in E_s$ and $e_2=\{u,v\} \in {\overline{E}}$ it cannot be that $\{v,s\} \not \in E_s$. It follows that there cannot exist a wedge $({e_1}\wedge {e_2})$ such that $e_1 \in E_s$ and $e_2 \in {\overline{E}}$.

Let $S\subseteq E_s$ be the edges in $E_s$ that are labeled strong according to $\ell ^{\prime }$. Each edge in $E_s$ corresponds to a vertex $v\in V$.

We can see that a pair of selected edges $e_1=\{s,v\}$, $e_2=\{s,u\}$ contributes to the objective function of the MaximizeSTCBridges-*d* problem if and only if $\{u,v\} \notin {\overline{E}}$ and, by construction of *H*, this happens if and only if $\{u,v\} \in E$. Note also that the MaximizeSTCBridges-*d* problem asks to select *k* edges in *H*, which correspond to *k* vertices in the original graph *G*. Therefore, the number of $\textsc {stc}$ bridges in *H*, induced by a solution to the MaximizeSTCBridges-*d* problem is at least *c* if and only if the corresponding *k*-subgraph in *G* has density at least *c*.

Additionally, given a labeling $\ell ^{\prime }$ we can verify in polynomial time whether it is a feasible solution for the MaximizeSTCBridges-*d* problem. Therefore, MaximizeSTCBridges-*d* is $\mathbf {NP}$-complete. $\square$

Regarding approximability, using the same construction as in the proof of Lemma 1, we can see that a *c*-approximate solution for MaximizeSTCBridges is also a *c*-approximate solution for the $\textsc {d}k\text {s}$ problem. However, the $\textsc {d}k\text {s}$ problem has been shown to not admit a $\textsc {ptas}$, and the best known approximation ratio to date is $\mathcal {O} (n^{\frac{1}{4}+\epsilon })$ and is due to Bhaskara et al. (2010).

Despite this negative result, in the following sections we show how to obtain a constant-factor approximation guarantee, in polynomial time, for certain special cases of interest.

## Connection with the densest *k*-subgraph problem

In the previous section, in order to prove the hardness of the MaximizeSTCBridges problem, we reduced the densest *k*-subgraph ($\textsc {d}k\text {s}$) problem to it. In this section we will delve further into the connection between the two problems, which is a key component of our algorithmic results. Our approach for solving the problem involves the following pipeline: First we transform the input graph into an appropriately constructed *wedge graph*, which maps the problem into a maximum-density finding problem. Then our solution for the MaximizeSTCBridges problem is obtained by solving a novel variant of the $\textsc {d}k\text {s}$ problem, which we call the *densify k*-*subgraph* ($k{{\text {-}}\textsc {Densify}}$) problem, on the wedge graph.

In Sect. 5.1 we present the $k{{\text {-}}\textsc {Densify}}$ problem. In Sect. 5.2 we demonstrate how to use the $k{{\text {-}}\textsc {Densify}}$ problem to solve the MaximizeSTCBridges problem, through an appropriately constructed *wedge graph*. In the next section we demonstrate how to solve the $k{{\text {-}}\textsc {Densify}}$ problem by adapting an existing algorithm for the $\textsc {d}k\text {s}$ problem, proposed by Chen et al. (2010), which we briefly describe in Sect. 5.3.

### Densified *k*-subgraph

The $k{{\text {-}}\textsc {Densify}}$ problem is a variant of $\textsc {d}k\text {s}$, where in addition to the graph $G$, we also receive as input a set of fixed vertices $F$, and the goal is to find an additional set $S$ of *k* vertices that maximize the density of the subgraph induced by the fixed vertices and the newly-selected *k* vertices. The fixed vertices model the presence of strong edges in the instance of MaximizeSTCBridges, which cannot be changed, but still induce $\textsc {stc}$ bridges.

#### Problem 3

($k{{\text {-}}\textsc {Densify}}$) Given a graph $G =(V,E)$ and a subset of vertices $F \subseteq V$, find a subset of vertices $S \subseteq V$ such that $S \cap F = \emptyset$, $|S | = k$, and the density $\rho (F \cup S)$ of the subgraph induced by the set of vertices $F \cup S$ is maximized.

As one may expect, Problem 3 is $\mathbf {NP}$-hard.

#### Proposition 1

The $k{{\text {-}}\textsc {Densify}}$ problem is $\mathbf {NP}$-hard. Furthermore, it does not admit a $\textsc {ptas}$.

#### Proof

It is easy to see that $\textsc {d}k\text {s}$ is a special case of $k{{\text {-}}\textsc {Densify}}$ (by assuming $F = \emptyset$). Furthermore, any approximation algorithm for $k{{\text {-}}\textsc {Densify}}$ can be used as an approximation algorithm for $\textsc {d}k\text {s}$ with the same approximation guarantee. It has been shown that the $\textsc {d}k\text {s}$ problem does not admit a $\textsc {ptas}$ (Bhaskara et al. 2010). $\square$

### The wedge graph

In this section we discuss how to apply $k{{\text {-}}\textsc {Densify}}$ in order to solve the MaximizeSTCBridges problem. Our mapping involves constructing a *wedge graph* $\mathcal {W}$ based on the input graph and solving the $k{{\text {-}}\textsc {Densify}}$ problem on the wedge graph. The concept of the wedge graph was also used by Sintos and Tsaparas in their work of inferring link types in social networks (Sintos and Tsaparas 2014).

We first present the construction of the wedge graph. Given an input graph $G =(V,E)$ consider the set of wedges $W$ of $G$, as defined in Sect. 3, i.e., a wedge is a relation between two edges that share a vertex while the “third” edge is not present. The wedge graph of $G$ is a graph $\mathcal {W} =(E,W)$ whose set of vertices are the edges $E$ of $G$, and whose set of edges are the wedges $W$ of $G$.

**Fig. 3:**
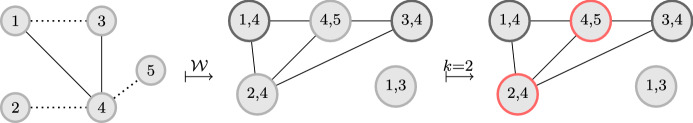
High-level description of our algorithmic pipeline using the example of Fig. 1 and for $k=2$. On the left hand side is the initial graph. Then, the graph is transformed into the corresponding wedge graph. Edge-vertices (1, 4) and (3, 4) are highlighted in black since they correspond to fixed vertices in the $k{{\text {-}}\textsc {Densify}}$ instance (edges (1, 4) and (3, 4) are strong). The final step is to obtain the optimal $k{{\text {-}}\textsc {Densify}}$ solution for $k=2$ (vertices in red) (Color figure online)

To solve the MaximizeSTCBridges problem on the input graph $G$ with a given edge labeling $\ell$, we construct the wedge graph $\mathcal {W}$ of $G$ and we take the set of fixed vertices $F$ of $\mathcal {W}$ to be the set of edges of $G$ with strong ties, i.e., $F =\left\{ e\in E \mid \ell (e)=\texttt {S} \right\}$. We then solve the $k{{\text {-}}\textsc {Densify}}$ problem on $\mathcal {W}$ with this set of fixed vertices $F$, and seeking to find *k* vertices in $\mathcal {W}$ (i.e., *k* edges in $G$). An illustration of this pipeline is shown in Fig. 3.

The solution set $S$ gives a solution for MaximizeSTCBridges on $G$: the selected *k* vertices maximize the density in $S$. Since the vertices in $\mathcal {W}$ correspond to edges in $G$ and the edges in the wedge graph correspond to wedges in $G$, the maximum-density subgraph on the selected vertices in $\mathcal {W}$ corresponds to the set of edges in $G$ that , when relabeled to strong edges in $\ell '$, maximize the number of $\textsc {stc}$ bridges.

Furthermore, if $S$ is a *c*-approximate solution for the $k{{\text {-}}\textsc {Densify}}$ problem, it is also a *c*-approximate solution for the MaximizeSTCBridges problem.

### Densest *k*-subgraph algorithm for graphs with $\sigma$-quasi-elimination order

A key concept that will be used in our algorithm is the notion of a $\sigma$-quasi-elimination order. The concept of $\sigma$-quasi-elimination orders was proposed by Akcoglu et al. (2002) as a generalization of perfect elimination orders for chordal graphs. Before formally introducing $\sigma$-quasi-elimination orders we introduce some preliminaries.

Let $$\alpha (G)$$ be the *independence number* of the graph $$G$$, i.e., the size of a maximum independent set in $$G$$. Let *N*(*v*) be the set of neighbors of vertex *v*, i.e. $$N(v)=\{u \mid \{v,u\} \in E \}$$. Recall that we denote by $$G [S ]$$ the subgraph of $$G$$   induced by the vertices of $$S $$. If $$\mathcal {L} =(v_1,\ldots ,v_n)$$ is an ordering of the vertices in $$V$$, we define $$\text {succ} _{\mathcal {L}} (v_i)=\{v_j \mid j>i \text { and } v_j \in N(v_i)\}$$ the set of *successors* of $$v_i$$, and $$\text {pred} _{\mathcal {L}} (v_i)=\{v_j \mid j<i \text { and } v_j \in N(v_i)\}$$ the set of *predecessors* of $$v_i$$. In a perfect elimination order, every set $$\text {pred} _{\mathcal {L}} (v_i)$$ is a clique. A $$\sigma $$-quasi-elimination order generalizes this definition by relaxing the requirement of having a complete clique.

#### Definition 3

($\sigma$-quasi-elimination order) Let $G =(V,E)$ be a graph and $\sigma$ a positive integer. A $\sigma$-quasi-elimination order ($\sigma {\textsf {\small -QEO}}$) of $G$ is an ordering $\mathcal {L}$ of the vertices $V$ such that $\alpha (G [\text {pred} _{\mathcal {L}} (v_i)])\le \sigma$, for all $i=2,\ldots ,n$.

We now present the algorithm of Chen et al. (2010) for the $\textsc {d}k\text {s}$ problem. The main result of Chen et al. is an $\mathcal {O} (\sigma )$-approximation algorithm for the $\textsc {d}k\text {s}$ problem if the input graph has a polynomial-time computable $\sigma$-quasi-elimination order.

The algorithm of Chen et al. relies on the *maximum-density subgraph problem* ($\textsc {mdsp}$) as a key subroutine. The maximum-density subgraph problem is defined as follows: given a graph $G=(V,E,w)$ with non-negative vertex weights $w : V \rightarrow \mathbb {R} _{\ge 0}$, we ask to find an induced subgraph $H=(V_H,E_H)$ maximizing the density

$$\begin{aligned} \rho (H)=\frac{\sum _{v\in V_H} w(v) + |E_H| }{|V_H|}. \end{aligned}$$

This problem can be solved optimally in $\mathcal {O} (nm\log (\frac{n^2}{m}))$ time by a reduction to the parametric maximum-flow algorithm (Gallo et al. 1989, Theorem 2.7). The reduction was introduced by Goldberg (1984).

The first step is to solve $$\textsc {mdsp}$$ on the graph *G* with weights $$w(v)=0$$, for all $$v \in V$$, and obtain a subgraph *H*. Let $$k'$$ be the number of vertices of *H*. If $$k' < k $$ then we repeat the $$\textsc {mdsp}$$ algorithm on the remaining vertices of *G* and combine the solution with *H*. This is Phase 1 of the algorithm, in which we iteratively remove vertices, while keeping track of the number of removed vertices by updating vertex weights in the next call to the $$\textsc {mdsp}$$ subroutine. If on the other hand $$k'>k$$, then we are in Phase 2 and some vertices from the obtained solution need to be removed, without losing too much in terms of density.

In the next section, we will adapt the algorithm by Chen et al. for the $\textsc {d}k\text {s}$ problem to obtain an algorithm for the $k{{\text {-}}\textsc {Densify}}$ problem, for which the following proposition holds.

#### Proposition 2

Let $$G =(V,E)$$ be a graph with edge labeling $$\ell $$, and let $$\mathcal {W} =(E,W)$$ be the wedge graph of $$G $$. If $$\mathcal {W} $$ has a polynomially-time computable $$\sigma {\textsf {\small -QEO}} $$, then we can obtain an $$\mathcal {O} (\sigma )$$-approximation for the MaximizeSTCBridges problem on graph $$G $$. The running time of the algorithm is $$\mathcal {O} (\sigma n^{\sigma +2})$$.

## Proposed algorithm

Our main result in this section is to show that the algorithm of Chen et al. (2010) can be carefully modified in order to solve the $k{{\text {-}}\textsc {Densify}}$ problem. The resulting algorithm, which we call *sigma-quasi-densify* (SQD), has the same $\mathcal {O} (\sigma )$-approximation guarantee for the $k{{\text {-}}\textsc {Densify}}$ problem as the algorithm of Chen et al. for the $\textsc {d}k\text {s}$ problem.

Now we will describe the main idea behind the modifications of the algorithm, to obtain SQD. Recall that in the $k{{\text {-}}\textsc {Densify}}$ problem we are given an input graph $G =(V,E)$ and a subset of vertices $F \subseteq V$, and the goal is to find a subset of *k* vertices $S \subseteq V$ such that the density $\rho (F \cup S)$ of the subgraph induced by the set of vertices $F \cup S$ is maximized.

Let us consider such a solution $$S $$ to an instance of the $$k{{\text {-}}\textsc {Densify}}$$ problem. The density is $$\rho =\frac{|E (S)|+|E (F)|+|E (S,F)|}{|S |+|F |}$$, where $$E (S)$$ are the edges contained in the induced subgraph $$G [S ]$$, $$E (F)$$ are the edges contained in the induced subgraph $$G [F ]$$, and $$E (S,F)$$ are the edges connecting vertices in $$S $$ and $$F $$. Note that the quantities $$|S |$$, $$|F |$$, and $$|E (F)|$$ are constant, so we can measure the performance of the algorithm with respect to the number of edges in $$E (S)\cup E (S,F)$$.

To ease the burden of notation, from now on we will write $E '=E (S)\cup E (S,F)$, that is, we disregard the edges among vertices in $F$ when we refer to edges in $E '$. In addition, we redefine density to be $\rho '=\frac{|E (S)|+ |E (S,F)|}{|S |}$. We will refer to the maximum possible value of $\rho '$ as $\rho ^*$ and a corresponding set of edges resulting in this density as $E ^*$. We can also see that any *c*-approximate solution $\rho '$ of $\rho ^*$ is also at least *c*-approximate for $k{{\text {-}}\textsc {Densify}}$.

As we will see in more detail below, during Phase 1 of the SQD algorithm we handle the vertices in $F$ by introducing an earlier step before the iterative calls to $\textsc {mdsp}$, where the fixed vertices are removed and the vertex weights are adjusted accordingly. In Phase 2, we compute a $\sigma$-quasi-elimination order for an appropriately constructed graph, which accounts for the vertices in $F$. By using this newly constructed graph we can prove the following.

### Proposition 3

There exists a $\mathcal {O} (\sigma )$-approximation algorithm for the $k{{\text {-}}\textsc {Densify}}$ problem, if the input graph has a polynomially-time computable $\sigma {\textsf {\small -QEO}}$.

We now describe in detail the two phases of our proposed algorithm SQD.

Phase 1: The first phase of the algorithm proceeds by iterative calls to an $$\textsc {mdsp} $$ subroutine. We introduce a preprocessing step compared to Chen et al. (2010), in which we remove the vertices in $$F$$. In particular, we define $$G_0=(V _0, E _0, w_0)$$ such that $$V _0=V \setminus F $$, $$E _0= E \setminus (E (F)\cup E (V _0,F))$$ and $$w_0(v)=|E (v,F)|$$ for all $$v \in V _0$$. The rest of Phase 1 proceeds in the same way as in the algorithm of Chen et al. (2010): starting with $$i=0$$ we find an optimal solution $$H_i=(V _{H_i},E _{H_i})$$ of density $$\rho '_i$$ by running $$\textsc {mdsp} $$ on $$G_i=(V _i, E _i, w_i)$$ with $$w_0(v)=|E (v,F)|$$ and $$w_i(v)=|E (v,U_{i-1} \cup F)|$$ in the case $$i>0$$, for $$v \in V _i$$, where $$U_i=\cup ^i_{j=0} V _{H_j}$$ is the set of so far all removed vertices (without the vertices in $$F $$, and therefore $$U_0=\emptyset $$), and $$n_i=|U_i|$$. Then we form graph $$G_{i+1}$$ by removing the vertices and incident edges of $$H_i$$ from $$G_i$$. We stop at the first time *t* such that $$n_t\ge \frac{k}{2}$$. If $$n_t \le k$$, then $$U_t$$ is returned along with some arbitrary $$k-n_t$$ vertices *Z* from $$V_{t+1}$$ as an approximate solution to the $$k{{\text {-}}\textsc {Densify}}$$ problem. Otherwise, if $$n_t >k$$ we proceed to Phase 2.

We adapt Lemma 4 from Chen et al. (2010), by accounting for the removed vertices from $$F $$, to prove that the process described above yields a 4-approximation algorithm.

### Lemma 2

(Chen et al. 2010, Lemma 4) If $n_t \le k$, the set $U_t \cup Z$ is a 4-approximation solution for the $k{{\text {-}}\textsc {Densify}}$ problem with input graph *G*.

The proof of Lemma 2 and all other missing proofs are provided in the Appendix, for better readability.

Phase 2: In this case we have $n_t > k$ and we must delete some vertices from the solution. In order to remove vertices while retaining an approximation guarantee for the quality of the solution, we will use the concept of $\sigma$-quasi-elimination orders, which we introduced in the previous section. Recall that a $\sigma$-quasi-elimination order ($\sigma {\textsf {\small -QEO}}$) of $G$ is an ordering $\mathcal {L}$ of the vertices $V$ such that $\alpha (G [\text {pred} _{\mathcal {L}} (v_i)])\le \sigma$, for all $i=2,\ldots ,n$. As we will see in Sect. 8.1, computing a $\sigma$-quasi-elimination order can be done in time $\mathcal {O} (\sigma ^2 n^{\sigma +2})$, using the algorithm presented by Ye and Borodin (2009).

In order to make Phase 2 work similarly to the algorithm of Chen et al. (2010), we need to make a slight modification. Recall that Phase 1 results in a set of vertices $U_t$. We define $J=G [U_t]$ as the subgraph induced by vertex set $U_t$. We add some vertices and edges to the induced subgraph *J* to obtain graph $J'$. In particular, we construct $J'$ by introducing $|E (v,F)|$ dummy vertices for each vertex $v \in U_t$ and connecting each one of these dummy vertices with *v*, through an edge. Then, we compute a $\sigma$-quasi-elimination order $\mathcal {L}$ for $J'$. Observe that we can choose $\mathcal {L}$ in such a way that the new dummy vertices come after the vertices in $U_t$, and hence $\mathcal {L}$ has a prefix that is a $\sigma$-$\textsf {\small QEO}$ for *J*.

A key observation is that in an optimal $\textsc {mdsp}$ solution $H_t$ with density $\rho '_t$, it holds for every vertex $v\in H_t$ that $w(v)+ deg _H(v) \ge \rho '_t$, for otherwise we could delete this vertex to obtain an induced subgraph of higher density. Based on this observation, we have that for any vertex *v* it is $|\text {succ} _{\mathcal {L}} (v)| \ge \frac{\rho '_t}{2}$ or $|\text {pred} _{\mathcal {L}} (v)| \ge \frac{\rho '_t}{2}$.

Following Chen et al. (2010), we discern two cases. The first case occurs if there exists a vertex $v\in U_t$ with $|\text {pred} _{\mathcal {L}} (v)|\ge \frac{k}{2}$ (Lemma 5). In this case, the predecessor set is large enough to allow us to to efficiently find a subgraph of $\frac{k}{2}$ vertices that is a $\mathcal {O} (\sigma )$-approximation for $\textsc {d}k\text {s}$. If no vertex in $\mathcal {L}$ has a predecessor set of size at least $\frac{k}{2}$, then Lemma 6 of Chen et al. ensures a $\mathcal {O} (\sigma )$-approximation. In Appendix A.1 we show how to obtain bounds analogous to the ones given by Lemmas 5 and 6 of Chen et al. (2010). Note that the proofs need to be modified to work for $k{{\text {-}}\textsc {Densify}}$.

### Lemma 3

(Chen et al. 2010, Lemma 5) If there is a vertex $v \in U_t$ with $|\text {pred} _{\mathcal {L}} (v)| \ge \frac{k}{2}$, then we can efficiently find a subgraph of $\frac{k}{2}$ vertices in $\text {pred} _{\mathcal {L}} (v)$, which is a $\mathcal {O} (\sigma )$-approximation solution for $k{{\text {-}}\textsc {Densify}}$ on *G*.

### Lemma 4

(Chen et al. 2010, Lemma 6) If there is no vertex $v\in U_t$ with $|\text {pred} _{\mathcal {L}} (v)| \ge \frac{k}{2}$, then we can efficiently find a subset of $U_t$ of size at most *k*, which is a $\mathcal {O} (\sigma )$-approximation solution for $k{{\text {-}}\textsc {Densify}}$ on *G*.

Thanks to our mapping between $k{{\text {-}}\textsc {Densify}}$ and MaximizeSTCBridges, which utilizes the wedge graph $\mathcal {W}$, it is trivial to convert the output of the SQD algorithm in order to obtain a labeling $\ell '$ that is a solution to MaximizeSTCBridges. To construct $\ell '$ from $\ell$, it suffices to set $\ell '(e)=\texttt {S}$ for all $v_e \in U_t$, which is the set of vertices of the wedge graph $\mathcal {W}$ returned by SQD.

Pseudocode for our method is given in Appendix A.2 as Algorithm 1.

*Running Time:* The running time of Phase 1 is due to the iterative calls to the $\textsc {mdsp}$ subroutine and is $\mathcal {O} (nm\log (\frac{n^2}{m}))$, while Phase 2 is dominated by the computation of a $\sigma$-$\textsf {\small QEO}$. The fastest known algorithm to compute a $\sigma$-$\textsf {\small QEO}$ is due to Ye and Borodin (2009) and requires $\mathcal {O} (\sigma n^{\sigma +2})$. We should also note that in the algorithmic pipeline we have described earlier, we run this algorithm on an $\mathcal {O} (m^2)$ wedge graph. Although this asymptotic running time may seem prohibitive for practical applications, these bounds are loose, and as we will see in the following sections, in practice we do not need to run most of the subroutines of the algorithm on really large graphs.

## Properties of the wedge graph

We now take a closer look at properties of the wedge graph and we derive conditions that lead to better approximation guarantees for our method. We will investigate the properties of the wedge graph with respect to $\sigma$-quasi-elimination orders. Before proceeding, we introduce some conventional notation when referring to cliques in graph theory. Let $K_t$ denote a clique of size *t*, while a *bi-clique* $K_{t,t}$ is a complete bipartite graph $G (U,V,E)$ where $|U|=|V|=t$. We refer to the *clique number* $\omega (G)$ as the maximum $t'$ such that $K_{t'}\subseteq G$. Apart from this standard notation, for convenience we will call two $K_t$ cliques with only one common vertex a *t*-*bowtie *.

First, we consider upper bounds for $\sigma$ of an optimal $\sigma$-$\textsf {\small QEO}$ for the wedge graph $\mathcal {W}$. We first present a (naïve) upper bound on $\alpha (\mathcal {W} [N(v_e)])$ for all vertices $v_e$ of $\mathcal {W}$.

### Proposition 4

Let $G =(V,E)$ be a graph and $\mathcal {W} =(E,W)$ its wedge graph. For all vertices $v_e\in E$ of the wedge graph it holds that $\alpha (\mathcal {W} [N(v_e)])\le 2(\omega (G)-1)$.

### Proof

Consider an edge $e=\{u, v\}$ of $G$ and the corresponding vertex $v_e$ of $\mathcal {W}$. Denote by $E _u$ the set of edges incident to vertex *u*, and $E _v$ the set of edges incident to vertex *v*. Consider first vertex *u*. Assume that there are $m_u\le |E _u|$ edges such that $N_G(u) \setminus N_G(v) = m_u$ (they do not form form a triangle with *e*). These edges form a wedge with *e*, and therefore the vertices in $\mathcal {W}$ corresponding to these edges will all be connected with an edge to $v_e$ in $\mathcal {W}$. Additionally, the edges in $E _u$ that pairwise form triangles with a third edge not in $E _u$, will not be connected with an edge in $\mathcal {W}$. We can see that the maximum independent set of vertices in $\mathcal {W}$ corresponding to edges in $E _u$ is formed when the endpoints of the edges outside of *u*, form a clique. Since the largest clique in $G$ is $\omega (G)$, and since the case for *v* is symmetric, $\alpha (\mathcal {W} [N(v_e)])$ is at most $2(\omega (G)-1)$. $\square$

As a consequence we obtain the following lemma.

### Lemma 5

If $G$ is $K_t$-free, then SQD gives a $(2t-4)$-approximation guarantee.

### Proof

If $G$ is $K_t$-free, then for all vertices $v_e \in E$ of the wedge graph $\mathcal {W}$ we have $\alpha (\mathcal {W} [N(v_e)])\le 2t-4$. Any arbitrary quasi-elimination order $\mathcal {L}$ will result in $\alpha (\mathcal {W} [\text {succ} _{\mathcal {L}} (v_e)])\le 2t-4$, therefore $\sigma \le 2t-4$. $\square$

For example, if $G$ is triangle-free, we obtain a 2-approximation, while if $K_3$ is the largest clique of *G* then we have a 4-approximation.

We can see that this bound is not very tight, since a large graph may have a high clique number. Additionally, even the presence of a large clique does not necessarily imply a large lower bound for the value of $\sigma$ for which a $\sigma$-quasi-elimination order of $\mathcal {W}$   exists. As an example, consider the wedge graph $\mathcal {W}$ of a graph that has one (or more) cliques $K_t$, with $t\ge 3$, but without two distinct $K_t$ cliques sharing a common vertex. Then the wedge graph $\mathcal {W}$ has a $\sigma$-quasi-elimination order with $\sigma =2$.

In the following we will describe a particular subgraph that appears in all graphs for which a $\sigma$-quasi-elimination order exists.

First, we have the following theorem related to $\sigma$-quasi-elimination orders (proof in Appendix A.1). The theorem presents an upper bound on the $\sigma$-quasi-elimination order of a subgraph *G* induced by a set of vertices *S*, based on the cardinality |*S*|.

### Theorem 1

Let $G =(V,E)$ be a graph that does not have a $\sigma$-quasi-elimination order with $\sigma \le t$, for some value $t \le n$. Then there exists a connected subgraph of $G$ induced by a minimal set of vertices $S \subseteq V$, such that $\alpha (G [S ]) > t$, and additionally $|S |\ge 2t$.

An example of such a minimal subgraph such that $G$ does not have a *t*-$\textsf {\small QEO}$ is the $K_{t,t}$ bi-clique. We can see that in a $K_{t,t}$ bi-clique all vertices *v* have $\alpha (G [v \cup N(v)])=t$, therefore there cannot be a possible elimination ordering of the vertices that produces $\alpha (G [u \cup N(u)]<t$ for any $u \in K_{t,t}$.

The previous results indicate that if a graph does not have a $\sigma$-quasi elimination order such that $\sigma \le t$, there must be a 2*t*-star present in $G$ with all edges incident to a $K_t$ clique in only one endpoint. However, here we will prove the following claim about the wedge graph $\mathcal {W}$ (proof in Appendix A.1).

### Theorem 2

If $\mathcal {W}$ does not have a 2*t*-$\textsf {\small QEO}$ then $G$ contains two $K_t$ cliques overlapping in only one vertex.

Theorem 2 can help us bound the value of the $\sigma$ for which a $\sigma$-$\textsf {\small QEO}$ exists, in cases where the maximum degree of $G$ is also bounded. Namely, we have the following lemma as a consequence.

### Lemma 6

The wedge graph $\mathcal {W}$ of a graph $G$ with maximum degree $\Delta$ has a quasi-elimination order with $\sigma \le \Delta$.

### Proof

Since the maximum degree is $\Delta$, each bowtie can consist of at most two $K_{\Delta /2}$ cliques (see proof of Theorem 2), therefore the graph has at most a $\Delta$-$\textsf {\small QEO}$. $\square$

Based on the observations in this section, in the following section we introduce two problem variants with constant factor-approximation guarantees.

## Constant-factor approximation for special cases of interest

In this section we present two special cases of the MaximizeSTCBridges problem. We show that in both cases the proposed approach provides a constant-factor approximation guarantee. Both special cases restrict the family of input graphs or the choices for the output, yet the restricted problem formulations are motivated by realistic scenarios.

### Graphs with bounded maximum degree

The strengthening of a social connection is a process that requires time and effort by both participants. Additionally, we would like to avoid overwhelming users with too many recommendations and potentially harming their experience. In the first special case we make the assumption that for each vertex we consider only the strongest (but still weak) connections to make stronger. Therefore, we consider strengthening a tie only if it belongs in the top-*d* weak ties of both vertices. Notice here that we assume that we are able to rank all the edges incident to a vertex in order of their strength. The top-*d* weak ties of a vertex *v*, ordered by their strength, are denoted by $P_d (v)$. The set of weak edges that belong in the $P_d$ list of both of their endpoints is denoted by $C_d = \{\{u,v\}\in E \mid {\ell (\{u,v\})=\texttt {W}} \text { and } \{u,v\}\in P_d (u)\cap P_d (v)\}$.

The problem we consider in this case is the following.

#### Problem 4

(MaximizeSTCBridges $_d$) Given a graph $G =(V,E)$ and a labeling function $\ell$ from the edges of $G$ to $\{\texttt {W},\texttt {S} \}$, find a labeling $\ell ^{\prime }$, which is a *k*-strengthening of $\ell$, and the number of $\textsc {stc}$ bridges ${\mathcal {B}} (\ell ^{\prime },G)$ induced by $\ell ^{\prime }$ on $G$ is maximized. Furthermore, the edges that are relabeled to $\texttt {S}$ by $\ell ^{\prime }$ are restricted to be in the set $C_d$.

For this restricted case we have the following approximability result.

#### Theorem 3

SQD returns a solution that is guaranteed to be a *d*-approximation to the MaximizeSTCBridges $_d$ problem.

The proof is an immediate consequence of the results in the previous section.

#### Proof

We saw that the wedge graph $\mathcal {W}$ of a graph $G$ with maximum degree *d* has a $\textsf {\small QEO}$ with $\sigma \le d$. It is easy to see that is the case for the restricted MaximizeSTCBridges $_d$ problem class. Therefore, SQD yields a factor-*d* approximation guarantee. $\square$

Additionally, we can also see that for this problem case we can compute a quasi-elimination order more efficiently.

Ye and Borodin (2009) studied the algorithmic properties of quasi-elimination orders. Among other results, they present an $\mathcal {O} (\sigma ^2n^{\sigma +2})$ algorithm for computing a $\sigma {\textsf {\small -QEO}}$. Here we show that in practice, we can do better than that.

#### Theorem 4

Let $G$ be a graph with *n* vertices, *m* edges, and maximum degree $\Delta$. A $\sigma {\textsf {\small -QEO}}$ can be computed in time $\mathcal {O} (\sigma ^2n\Delta ^{\sigma +1})$.

#### Proof

Our construction is similar to the one by Ye and Borodin. We build the bipartite graph $G^*=(A,B)$ as follows. We construct a subset-node in *A* for each subset of size $\sigma$ in $G$ and a vertex-node in *B* for each vertex in $G$. Observe that since the maximum degree in $G$ is $\Delta$, a vertex in $G$ is connected to at most $\Delta$ vertices. Therefore for each vertex we need only construct $\Delta ^{\sigma +1}$ subset-nodes in *A*. We connect a vertex-node to a subset-node with a red edge if the vertex in the vertex-node is adjacent to all vertices in the subset-node, and the vertices in the subset are independent. We connect a vertex-node to a subset-node with a black edge if the vertex in the vertex-node is one of the vertices in the subset-node. Constructing such a graph $G^*$ takes $\mathcal {O} (\sigma ^2n\Delta ^{\sigma +1})$. The algorithm is the same as described by Ye and Borodin leading to a total complexity of $\mathcal {O} (\sigma ^2n\Delta ^{\sigma +1})$.$\square$

Recall that in this case we consider $\Delta$ to be small and thus expect the algorithm to run in reasonable time. We further offer a practical speed up, by observing that we do not need to generate subset-nodes that are not incident to red edges. Notice that a subset-node can never be incident to a red edge if it is not an independent set. Additionally, since all subsets of independent sets are also independent, we can use an apriori-style algorithm, where we generate candidate sets of size *k* from independent sets of size $k-1$. As we will see in the experiments, this leads to an algorithm that is quite efficient in practice.

### Strengthening local bridges

We now propose another special case of the problem with a different aim—we want to leverage the $\textsc {stc}$ property to increase the number of connections between weakly connected parts of the social network. This can be beneficial in settings where the social network is fragmented into strong communities with a small number of connections between them. Connections between different communities has been shown to be facilitated by *local bridges*, which Easley and Kleinberg define as follows (Easley and Kleinberg 2010).

#### Definition 4

(Local bridge) An edge between two vertices *u* and *v* is called *local bridge* if *u* and *v* have no common neighbors.

Local bridges are important because they provide their endpoints with access to parts of the network, and hence sources of information, which would otherwise be far away.

Considering the above discussion we focus on strengthening local bridges. We aim to strengthen the local bridges that according to the $\textsc {stc}$ property will lead to the highest amount of new edges, thus increasing the number of connections between parts of the graph that are weakly connected.

Given a graph $G =(V,E)$, let $L \subseteq E$ be the local bridges of $G$.

#### Problem 5

(MaximizeLocalBridges) Given a graph $G =(V,E)$ and a labeling function $\ell$ from the edges of $G$ to $\{\texttt {W},\texttt {S} \}$, find a labeling $\ell ^{\prime }$, which is a *k*-strengthening of $\ell$, and the number of $\textsc {stc}$ bridges ${\mathcal {B}} (\ell ^{\prime },G)$ induced by $\ell ^{\prime }$ on $G$ is maximized. Furthermore, the edges that are relabeled to $\texttt {S}$ by $\ell ^{\prime }$ are restricted to be in the set of local bridges $L$.

Again for this case, we can show that our algorithm provides a constant-factor approximation guarantee.

#### Theorem 5

The MaximizeLocalBridges problem has a factor-2 approximation guarantee.

#### Proof

Observe that in the construction of the wedge graph $\mathcal {W}$ we can ignore all edges $e=\{v_1,v_2\}$ for which $\ell (e)=\texttt {W}$ and $|N(v_1)\cap N(v_2)|>1$, since they will not be considered in the in the set of edges to be relabeled by MaximizeLocalBridges, and they cannot create any $\textsc {stc}$ bridges. To obtain the solution we run SQD with input the wedge graph $\mathcal {W}$ and $F =\{e : \ell (e)=\texttt {S} \}$. Consider an edge $e=\{v_1,v_2\}$ corresponding to a vertex $v_e$ of $\mathcal {W}$ returned by SQD. In the wedge graph $\mathcal {W}$, a vertex $v_e$ corresponding to such edge *e* will be connected to all edge-vertices $v_{e'}$ where $e'=\{u,v_1\}, u \in N(v_1)$. If such an edge $e'$ is labeled $\texttt {W}$, then since it cannot be part of any triangles, in $\mathcal {W}$ it will be connected to all other edge-vertices in $\{\{u,v_1\}: u \in N(v_1)\}$. If such an edge $e'$ is labeled $\texttt {S}$ it will be included in the set of fixed vertices $F$, in the instance of $k{{\text {-}}\textsc {Densify}}$. However, recall that in SQD, vertices in $F$ do not have an impact on the optimal $\sigma {\textsf {\small -QEO}}$. The case for $N(v_2)$ is symmetric, and from this we can see that $\alpha (\mathcal {W} [v_e \cup N(v_e)])\le 2$. Therefore there always exists a $\sigma {\textsf {\small -QEO}}$ with $\sigma =2$, giving a factor-2 approximation. $\square$

## Experimental evaluation

The goal of the experiments is to evaluate the performance of the algorithms for the MaximizeSTCBridges problem, both in terms of the number of $\textsc {stc}$ bridges achieved, and the running time. The experiments are conducted on real data, and demonstrate the practical efficiency of the algorithms.

### Heuristics

The algorithm presented in the previous section is of theoretical interest, however, it is not always scalable to large graphs, due to the large worst-case complexity of the $\sigma$-$\textsf {\small QEO}$-computation step.

In this section, we consider alternative algorithms, which are simpler to implement and/or more efficient.

Next, we present two greedy algorithms for MaximizeSTCBridges, which scale linearly to the size of the input graph. Additionally, as we will see in our experimental evaluation, the greedy algorithms yield solutions of extremely high quality, in practice.

*Local heuristic* The first scalable algorithm is a heuristic (we will simply call it Heuristic). It greedily selects to strengthen the ties that are adjacent to the biggest number of strong ties, resulting in the biggest number of $$\textsc {stc}$$ bridges after strengthening a weak tie. It is a local heuristic because it only considers the local benefit of strengthening a weak edge, without adapting for the incremental benefit of strengthening multiple weak ties. We expect this heuristic to perform well for small values of $$k $$. Regarding the running time, we can find the number of adjacent strong edges in $$\mathcal {O} (m)$$, while we need $$\mathcal {O} (m+k \log m)$$ for the top-*k* computation, which is also the overall asymptotic running time of the algorithm.

*Greedy* As we discussed, the Heuristic algorithm has the drawback of selecting edges independently, ignoring the additive benefit of strengthening pairs of edges. Our second greedy algorithm (named Greedy) overcomes this drawback by selecting edges iteratively and evaluating the gain in the objective function for each new edge. The Greedy algorithm starts with the input $\ell$, and in each step finds an edge $\{u,v\}$, which $\ell (\{u,v\})=\texttt {W}$, and converts it to strong, $\ell ^{\prime } (\{u,v\})=\texttt {S}$. The edge is selected greedily, such that ${\mathcal {B}} (\ell ^{\prime },G)-{\mathcal {B}} (\ell ,G)$ is maximized. The algorithm continues strengthening edges while the total number of selected edges does not exceed the budget $k$. For a solution with at most *p* relabeled edges, the cost of selecting the best candidate in each iterative step is $\mathcal {O} (m p)$. With an efficient implementation, the total running time of the Greedy is $\mathcal {O} (m p^2)$. In typical scenarios we can assume $p \ll m$, making the algorithm very efficient.

### Datasets

For our experimental evaluation we use real-world datasets, where each edge represents a social relation between two individuals. We only consider weighted networks, where the edge weights correspond to an empirical strength of the connection. We use the edge weight as a proxy for tie strength, and in the following experiments, we arbitrarily pick the 70% percentile of edge strength as the separator between strong and weak ties. We assume that all weak ties can be converted to strong.

We use seven different datasets in our experiments: *LesMis*, *KDD*, *Facebook*, *Twitter*, *Telecoms*, *BitCoinAlpha*, and *Retweets*. The datasets first appeared in Adriaens et al. (2018) and Lahoti et al. (2018) and were kindly shared with us by the authors. The networks convey different types of social trust, and have been used in STC literature before. Table 1 shows some statistics about our datasets. The first two columns of the table contain the number of vertices and edges of each network, the following two the number of strong and weak edges, while the last column is the global clustering coefficient of the networks. If *T* is the number of triangles in a graph then the clustering coefficient is $C=\frac{T}{T+W}$.

**Table 1:** Dataset statistics

Dataset	Vertices	Edges	Strong	Weak	C
*LesMis*	77	254	72	182	0.498
*KDD*	2 738	11 073	3 159	7 914	0.162
*Facebook*	3 228	4 585	867	3 718	0.056
*Twitter*	4 185	5 680	1 694	3 986	0.007
*Telecoms*	8 665	12 132	3 218	8 914	0.002
*BitCoinAlpha*	3 775	14 120	2 506	11 614	0.078
*Retweets*	200 073	4 009 548	251 450	3 758 098	–

Distinction into strong and weak is based on the 70-percentile of ground-truth tie strengh

### Performance evaluation

**Fig. 4:**
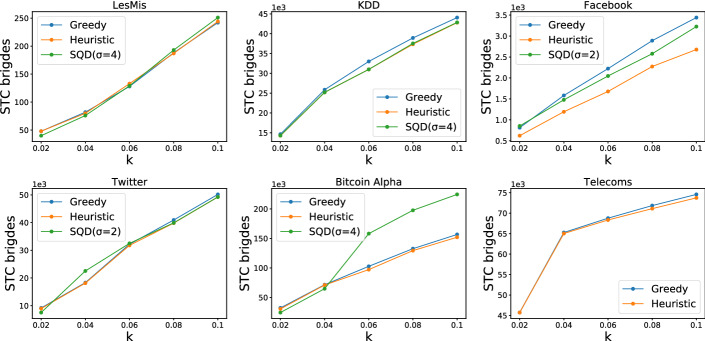
Performance comparison of all algorithms

We now proceed to evaluate the proposed algorithms with respect to the number of $\textsc {stc}$ bridges they achieve. The experiments were performed on a machine with 28 GB of RAM and 8 cores. SQD is the algorithm described in the previous sections, Greedy and Heuristic are the two greedy algorithms. In the case of SQD we also report (in parentheses) the lowest value of $\sigma$ for an elimination order, found during the execution of the algorithm. As noted before, this represents the approximation guarantee of the algorithm. Figure 4 shows the results obtained by the algorithms on all datasets (except *Retweets* where only Heuristic terminates within reasonable time), where *k* is reported as a fraction of the total number of edges *m*.

We observe that Greedy in most cases achieves the best performance, followed by SQD. In general the algorithms perform closely to each other, however SQD heavily outperforms the other two on the *BitCoinAlpha* dataset. We believe this may be due to the presence of a large dense component in the wedge graph of this dataset, which SQD is able to detect. Heuristic achieves a good performance for smaller values of $$k $$, due to picking first the edges that are adjacent to many strong edges, resulting in immediate benefit. For larger values of $$k $$, the effect of these edges vanishes. This is mostly evident in the fact that Heuristic is always the worst performing algorithm for $$k =0.07m $$ and larger. We remind that despite the fact that in the tested datasets SQD sometimes fails to achieve a better performance than Greedy, it has a performance guarantee, based on the optimal value of $$\sigma $$. Finally, the results also confirm our expectation of low optimal $$\sigma $$ values, in practice.

**Fig. 5:**
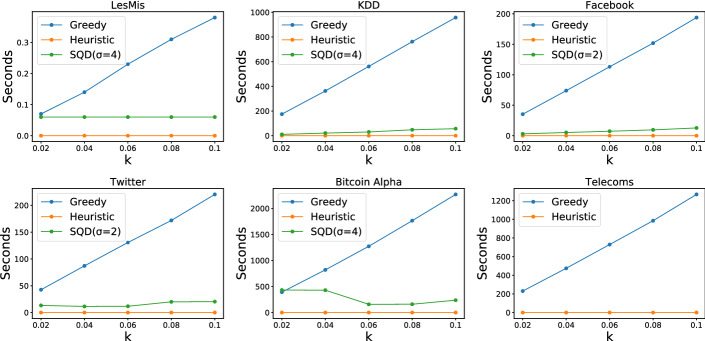
Running time comparison of all algorithms

### Scalability

We also perform a scalability analysis of the algorithms, with the results shown in Fig. 5. SQD was not able to terminate within reasonable time on *Telecoms* for any value of $$k $$. We can see that Greedy scales linearly with $$k $$, while Heuristic is very scalable since its complexity is logarithmic with respect to $$k $$. We note that Heuristic is capable of terminating fast even on *Retweets*, which has more than 4 M edges. On this dataset Heuristic terminated within 0.033 seconds and achieved $$1\,814\,583$$ potential new edges. Regarding SQD, we notice that it is relatively scalable in most instances and that it does not follow a trend with respect to $$k $$. Although the algorithm has a high worst-case complexity, which is dominated by the optimal $$\sigma $$ elimination computation step, this step is only applied on the subgraph returned from the first step of the algorithm, which is usually relatively small. We note that the size of the subgraph returned from the first step is dependent on the clustering coefficient of the initial graph; graphs with a low clustering coefficient contain many wedges, and lead to wedge graphs that are denser. For example in *Telecoms*, which has a very low clustering coefficient, the algorithm does not terminate within reasonable time.

## Limitations and discussion

In this section we discuss limitations of our work. Some of these limitations present challenges to overcome in order to fully realize the potential of our proposed framework, and should serve as a direction for future work.

First, as we noted earlier, our model is graph-driven rather than user-driven. In particular, we aim to utilize the structure of the social graph, while making minimal assumptions regarding user behavior. We only consider relationships between users and distinguish them between strong and weak ties. No other assumption is made about the nature of relationships between users. Accordingly, we have to assume that all weak ties can be converted into strong with equal difficulty. This is an oversimplifying assumption, since in practice not all weak ties are the same, and neither are all strong ties. In order to handle this issue, and given more data on the relationships between users, one could reason about a probabilistic model, where each tie is converted to strong with a certain probability. Note that since in our framework we consider a node-weighted variant of the $\textsc {d}k\text {s}$ problem, our solution can be easily adjusted to incorporate the edge-probabilities as node-weights in the wedge graph.

Another implication of our approach to distinguish all edges as only strong or weak, is that it prevents us from giving a specific description of a tie-strengthening mechanism, as such a mechanism would require additional knowledge about the specific nature of friendships in the social network. However, we can assume that such a mechanism is available in the form of a feature that the social network offers to the users to opt-in. We can then only consider strengthening edges whose both endpoints are users that have decided to opt-in to this feature. Note, that we can easily prune from the graph the rest of the edges, and implement our framework on the pruned graph.

It should also be kept in mind that our problem formulation, due to being simple and coarse-grained, can easily be fine-tuned to capture more nuanced cases. Our method describes an algorithm to strengthen edges in a social network instance, but is agnostic of the impact of social connections or how the given graph was created. One may preprocess the graph, for example, and remove all low-strength connections that have low chance of becoming strong (see Sect. 8), so as to focus only on the strongest (but still weak) connections. We believe that our framework offers many capabilities for such fine tuning, which can be incorporated as an additional preprocessing step in the input graph generation.

Another potential limitation of our work is that the proposed algorithm has a step with running time $\mathcal {O} (\sigma ^2n^{\sigma +2})$. Although this may appear infeasible in practice due to the exponentiated $\sigma$, in Sect. 7 we show that for high values of $\sigma$ to be possible, a very specific structure needs to appear in the graph (we call this structure a bowtie). Therefore, even for high-degree and power-law graphs it is unlikely that such large structures will emerge. We empirically demonstrate in the experiments that our algorithm is capable of running even on large datasets. However, developing a faster algorithm is an open line of research that has attracted considerable attention recently.

Finally, in order to minimize the disruption of the organic structure of the network, we consider a limit on how many ties can be converted from weak to strong, by introducing a budget *k*. However, this may still face the problem of overloading a single user with too many suggestions. In order to handle this, apart from the ideas mentioned in Sect. 8, one could consider an alternative formulation where a per-user budget *b* is considered. We note that such an alternative formulation is at least as hard as MaximizeSTCBridges. To see this, observe that in the reduction used in Lemma 1, the ego-network of a singe node *s* is sufficient to reduce to problem to $\textsc {d}k\text {s}$. In this case, the per-user budget *b* can be set to the global budget *k*.

## Conclusion

We considered the problem of leveraging the $\textsc {stc}$ property to introduce new edges in a social network. We formally defined the MaximizeSTCBridges problem, and we gave $\mathbf {NP}$-hardness and approximability results. We defined a novel variant of the well-studied $\textsc {d}k\text {s}$ problem, the $k{{\text {-}}\textsc {Densify}}$, which we map to MaximizeSTCBridges. This mapping leads to an approximation algorithm, and additionally allows us to prove various properties of the problem. Utilizing this insight, we define two problem variants that have a constant-factor approximation guarantee. Finally, in the experimental section we experiment with our algorithm in practice and we offer some scalable algorithms, which we evaluate on real data.

Our work opens several interesting directions for future work. A main challenge is to devise algorithms that are both scalable and have provable guarantee for the quality of the solution. Speeding up the algorithm of Ye and Borodin for computing quasi-elimination orders is another challenge towards the same end. Another direction is to explore different constraints regarding the problem of strengthening ties. The present formulation tends to bias a solution towards high degree nodes, which may be undesirable for the behavior of a content-recommendation algorithm. To counter-act this, one may impose a per-user budget for content recommendations.

Finally, another direction for future work is to deploy the proposed algorithm on a real-world social network and evaluate its performance on a practical setting.

## A Appendix

### A.1 Analysis of algorithms

For the analysis of our algorithms we use the following Lemma directly derived from the Turan bound (Chen et al. 2010). Assume that $n=|V |$ and $m=|E |$:

#### Lemma 7

(Turan bound) For any graph $$G $$, $$m \ge \frac{n^2-n\alpha (G)}{2\alpha (G)}$$

#### Proof (of Lemma 2)

Let $G^*(V^*,E^*)$ be an arbitrary optimal solution to the $k{{\text {-}}\textsc {Densify}}$ problem. In order to prove the lemma we need to reason about the edges of $G [(U_t\cap V ^*)\cup F ]$ minus the edges of $G [F ]$, since they are not factored in the optimal solution. There are two possible cases. If $|E ( (U_t\cap V ^*)\cup F)|-|E (F)| \ge \frac{|E ^*|}{2}$, then $U_t\cup Z$ is trivially a 2-approximation for $k{{\text {-}}\textsc {Densify}}$   (and hence also a 4-approximation, as the lemma requires). If not, then we define the sets $I_i=U_i \cap V^*$ and $R_i=V^* \setminus I_i$, for all *i*. We have that $|E ( (U_t\cap V ^*)\cup F)|-|E (F)|=|E (I_t\cup F)|-|E (F)| < \frac{|E ^*|}{2}$. Since $|E (I_t\cup F)| = |E (I_t)| + |E (I_t,F)| + |E (F)|$, we have that

$$\begin{aligned} |E (I_t\cup F)|-|E (F)| < \frac{|E ^*|}{2}, \end{aligned}$$

is equivalent to

$$\begin{aligned} |E (I_t)| + |E (I_t,F)| + |E (F)| - |E (F)| < \frac{|E ^*|}{2}, \end{aligned}$$

and thus,

$$\begin{aligned} |E (I_t)| + |E (I_t,F)| < \frac{|E ^*|}{2}. \end{aligned}$$

Additionally,

$$\begin{aligned} \rho '_i= & {} \frac{|E _{H_i}|+|E (U_{i-1}\cup F, V _{H_i})|}{|V _{H_i}|} \\\ge & {} \frac{|E (R_{i-1})|+|E (U_{i-1}\cup F, R_{i-1})|}{|R_{i-1}|} \\\ge & {} \frac{|E (R_{i-1})|+|E (I_{i-1}\cup F, R_{i-1})|}{|R_{i-1}|} \\\ge & {} \frac{|E (R_{i-1})|+|E (I_{i-1}, R_{i-1})|+|E (F, R_{i-1})|}{k}. \end{aligned}$$

Here we observe that all edges in $|E (F, R_{i-1})|$ belong to $E ^*$, so based on the above we can write:

$$\begin{aligned} \rho '_i\ge & {} \frac{|E ^*|-|E (I_{i-1})|-|E (F,I_{i-1})|}{k} \\\ge & {} \frac{|E ^*|-|E (I_t)|-|E (F,I_t)|}{k} \ge \frac{|E ^*|}{2k} =\frac{\rho ^*}{2} \\ \end{aligned}$$

We conclude that

$$\begin{aligned} |E (U_t)|+|E (F,U_t)| \;\ge \; \min _{i\le t} \left\{ \rho '_i |U_i| \right\} \;\ge \; \min _{i\le t} \left\{ \rho '_i \right\} \frac{k}{2} \;\ge \; \frac{|E ^*|}{4}. \end{aligned}$$

$$\square $$

#### Proof (of Lemma 3)

We define $\mathcal {A}=\text {pred} _{\mathcal {L}} (v)$. From the $\sigma$-quasi-elimination order property, and using Lemma 7, we conclude that the subgraph $G[\mathcal {A}]$ has at least $$\frac{1}{2\sigma }\left( {\begin{array}{c}|\mathcal {A}|\\ 2\end{array}}\right) $$ edges: We choose uniformly at random $\frac{k}{2}$ vertices from $\mathcal {A}$ to form $\mathcal {B}$. Let *e* be a given edge in $E (\mathcal {A})$. The probability that *e* is also an edge in the subgraph induced by the randomly-selected set of vertices $\mathcal {B}$ is

$$\begin{aligned} p_e = \mathbb {P}[e\in E (\mathcal {B})] = \frac{\frac{k}{2}}{|\mathcal {A}|} \frac{(\frac{k}{2}-1)}{(|\mathcal {A}|-1)}, \end{aligned}$$

where $\frac{\frac{k}{2}}{|\mathcal {A}|}$ is the probability that the first endpoint of *e* is in $\mathcal {B}$, and $\frac{\frac{k}{2}-1}{|\mathcal {A}|-1}$ is the probability that the second endpoint of *e* is in $\mathcal {B}$ given that the first endpoint is in $\mathcal {B}$. We define the random variable $X_e$ such that

$$\begin{aligned} X_e = {\left\{ \begin{array}{ll} 1 &{} \text {if } e \in E (\mathcal {B}), \\ 0 &{} \text {othersize}. \end{array}\right. } \end{aligned}$$

It follows that

$$\begin{aligned} |E (\mathcal {B})| = \sum _{e \in E (\mathcal {A})} X_e. \end{aligned}$$

The expectation of $X_e$ is

$$\begin{aligned} \mathbb {E}[X_e] = 1\cdot p_e + 0\cdot (1-p_e) = p_e, \end{aligned}$$

and thus,

$$\begin{aligned} {\mathbb {E}[|E (\mathcal {B})|] }&{=}&{\mathbb {E}\left[ \sum _{e \in E (\mathcal {A})} X_e\right] } \\&{=}&{\sum _{e \in E (\mathcal {A})} \mathbb {E}[X_e]} \\&{=}&{\sum _{e \in E (\mathcal {A})} p_e }\\&{=}&{\sum _{e \in E (\mathcal {A})} \frac{\frac{k}{2}}{|\mathcal {A}|} \frac{(\frac{k}{2}-1)}{(|\mathcal {A}|-1)}} \\&{=}&{|E (\mathcal {A})| \frac{\frac{k}{2}}{|\mathcal {A}|} \frac{(\frac{k}{2}-1)}{(|\mathcal {A}|-1)},} \end{aligned}$$

where the second equality is obtained by the linearity of expectation.

We now apply Lemma 7, which lower bounds $$|E(\mathcal {A})|\ge \frac{1}{2\sigma }\left( {\begin{array}{c}|\mathcal {A}|\\ 2\end{array}}\right) $$ and gives us:

$$\begin{aligned} \mathbb {E}[|E(\mathcal {B})|]\;\ge \; {\frac{1}{2\sigma }\frac{|\mathcal {A}|(|\mathcal {A}|-1)}{2}\frac{\frac{k}{2}}{|\mathcal {A}|} \frac{(\frac{k}{2}-1)}{(|\mathcal {A}|-1)}} \;=\; \frac{1}{\sigma } \left( \frac{k^2}{16}-\frac{k}{8}\right) . \end{aligned}$$

Notice that we can de-randomize the algorithm using the technique of conditional probabilities.

Additionally, we rank all vertices $v \in G$ according to $|E (v,F)|$ and pick the top $\frac{k}{2}$ vertices to form $\mathcal {C}$. We add the vertices in $\mathcal {C}$ to $\mathcal {B}$, to obtain $\mathcal {B'}$.

We can lower bound $|E (\mathcal {B'})|+|E (\mathcal {B'},F)|$ as

$$\begin{aligned} |E (\mathcal {B'})|+|E (\mathcal {B'},F)|&\ge \frac{1}{\sigma } \left( \frac{k^2}{16}-\frac{k}{8}\right) + |E (\mathcal {C},F)| \\&\ge \frac{1}{\sigma } \left( \frac{|E (V ^*)|}{16}-\frac{k}{8}\right) + \frac{|E (V ^*,F)|}{2} \\&\ge \frac{1}{\sigma } \left( \frac{k\rho ^*}{16}-\frac{k}{8}\right) \end{aligned}$$

We can see that the density of $\mathcal {B'}$ is $\rho '(\mathcal {B'})=\Theta \left( \frac{1}{\sigma }\rho ^*\right)$, which is a $\mathcal {O} (\sigma )$-approximation.

$$\square $$

#### Proof (of Lemma 4)

For each vertex *v* it holds that either $|\text {pred} _{\mathcal {L}}(v)| \ge \frac{\rho '_t}{2}$ or $|\text {succ} _{\mathcal {L}}(v)| \ge \frac{\rho '_t}{2}$. Note that $\text {succ} _{\mathcal {L}} (v)$ contains also the copy vertices from $F$. Since $\text {pred} _{\mathcal {L}} (v)$ does not contain any vertices from $F$, we can handle the case that $|\text {pred} _{\mathcal {L}}(v)| \ge \frac{\rho '_t}{2}$, in the same way as Chen et al. (Lemma 6).

We now present the proof of Chen et al., which also works for our case (due to the fact that $\text {succ} _{\mathcal {L}} (v)$ contains the copy vertices from $F$). We process the vertices of $U_t$ in the reverse order of $\mathcal {L}$, i.e., beginning at $v_{n_t}$. If a vertex *v* satisfies the condition $|\text {succ} _{\mathcal {L}}(v)| \ge \frac{\rho '_t}{2}$, we take just the vertex *v*, otherwise if it satisfies the condition $|\text {pred} _{\mathcal {L}}(v)| \ge \frac{\rho '_t}{2}$ we also take a certain subgraph of high-degree vertices of its predecessor set, along with it. To obtain this subgraph we set $\mathcal {A}=\text {pred} _{\mathcal {L}}(v)$ as the starting graph and we repeatedly delete a vertex of degree less than $\frac{|\text {pred} _{\mathcal {L}}(v)|-1}{4\sigma }$. By

$$\begin{aligned} |\text {pred} _{\mathcal {L}}(v)| \ge \frac{\rho '_t}{2} \ge \frac{\rho ^*}{4} \ge 2\sigma , \end{aligned}$$

it follows that the induced subgraph $G [\text {pred} _{\mathcal {L}}(v)]$ contains at least $$\frac{1}{2\sigma }\left( {\begin{array}{c}|\text {pred} _{\mathcal {L}}(v)|\\ 2\end{array}}\right) $$ edges, and thus, we cannot delete all vertices (and their edges) of $\text {pred} _{\mathcal {L}}(v)$. We stop if we have collected at least $\frac{k}{2}$ vertices. In every step, we add either a single vertex *v* or a subset of its predecessors to the solution. Since no vertex has a predecessor set of size at least $\frac{k}{2}$, we select at most *k* vertices in total, i.e., we obtain a feasible solution $\mathcal {B}$ for $k{{\text {-}}\textsc {Densify}}$.

Each vertex *v* in $\mathcal {B}$ has degree either at least $\frac{\rho '_t}{2}$, if it was selected by the first condition, or it holds that $\frac{|\text {pred} _{\mathcal {L}}(v)|}{4\sigma }\ge \frac{\rho '_t-2}{8\sigma }$, if it was selected by the second condition. Thus,

$$\begin{aligned} |E (\mathcal {B})|+|E (\mathcal {B},F)|&=\frac{1}{2}\sum _{v\in \mathcal {B}}|E (v,\mathcal {B})|+|E (v,F)| \\&\ge \frac{\rho '_t-2}{8\sigma }k \ge \frac{\rho ^*-4}{8\sigma }k \\&=\mathcal {O} \left( \frac{1}{\sigma }|E ^*|\right) . \end{aligned}$$

$$\square $$

#### Proof (of Theorem 1)

Let $$S \subseteq V $$. If there exists a vertex *v* in $$S $$ such that $$\alpha (G [v\cup N(v)]) \le t$$ then $$S$$ is trivially not minimal. Assume for contradiction that there exists a vertex $$v \in S $$ with $$\alpha (G [v\cup N(v)]) > t$$, such that $$\alpha (G [S ]) \le t$$ in the induced subgraph $$G [S ]$$. Then there exists an ordering $$\mathcal {L} $$ such that all $$u_i\notin S \cap N(v)$$ are selected before *v*, so that $$\alpha (G [\{v\} \cup \text {succ} _{\mathcal {L}} (v)])<t$$. Then we select *v*, and since $$S$$ is connected, this reduces $$\alpha (G [\{u\} \cup \text {succ} _{\mathcal {L}} (u)])$$ of at least one vertex *u* in $$S$$. Applying this inductively, we obtain a $$\sigma $$-elimination order of $$S$$ with $$\sigma \le t$$. However, this is a contradiction since we assumed that $$G$$ does not have a $$\sigma $$-elimination order such that $$\sigma \le t$$. Therefore this minimal set $$S$$ has $$\alpha (G [S ]) > t$$.

Additionally, each vertex $$v \in S $$ must be connected to at least *t* vertices in $$S$$, since $$S $$ is minimal and $$\alpha (G [S ]) > t$$. Then there exists a vertex $$u \in N(v)$$ such that $$|N(v)\setminus N(v) \cap N(u)| \ge t-1$$, otherwise we would have $$\alpha (G [\{v\} \cup S ]) \le t$$. This means that the set $$S$$ has *t* vertices that are not among the *t* neighbors of *v*, therefore $$|S |\ge 2t$$. $$\square $$

#### Proof (of Theorem 2)

Recall that a *t*-bowtie is a set of two $K_t$’s with a single common vertex. First note that if $G$ does not contain a *t*-bowtie, then it also does not contain a $t'$-bowtie, with $t'>t$, since a *t*-bowtie is a subgraph of the $t'$-bowtie.

Now assume for contradiction that $G$ does not contain a *t*-bowtie. In the simple case that the graph $G$ does not contain any $K_t$ clique, then it is easy to see that in $\mathcal {W}$ there cannot exist a vertex $v_i$ with $\alpha (\mathcal {W} [v_i\cup N(v_i)]) \ge t$, since there do not exist *t* pairwise disconnected vertices.

So now we consider the case that $G$ contains $K_t$ cliques but they do not share any vertices. Then for an edge *e* in $G$, it is either adjacent to a $K_t$ clique or the corresponding vertex $v_e$ in $\mathcal {W}$ has $\alpha (v_e)< t$. If it is adjacent to a $K_t$ clique then $t \le \alpha (\mathcal {W} [v_e\cup N(v_e)]) \le 2t$, however all adjacent edges $e' \in K_t$ have $\alpha (\mathcal {W} [v_e'\cup N(v_e')]) \le 2(t-1)$, since they are not adjacent to any $K_t$. Therefore in an elimination order $\mathcal {L}$ for $\mathcal {W}$ they can be selected before *e*, reducing its sequential order number by *t*. Therefore the optimal $\sigma$-quasi-elimination has $\sigma < 2(t-1)$, which is a contradiction.

Now assume that the graph $G$ contains two $K_t$ cliques, with the edges that take part in the $K_t$ cliques denoted as $E _c$, and there exists an edge $e'$ that forms triangle with two edges from $E _c$. Then in the wedge graph $\mathcal {W}$ we have $d(v_e) < 2(t-1)$, for $e \in E _c$, and therefore $\alpha (\mathcal {W} [v_e\cup N(v_e)]) < 2(t-1)$.

In all cases we can see that there cannot exist an optimal $\sigma$-quasi-elimination with $\sigma \ge 2t$. $\square$

### A.2 Pseudocode for SQD algorithm

For clarity we provide pseudocode for the SQD algorithm, as Algorithm 1.

## Footnotes

1 https://www.washingtonpost.com/technology/2021/10/26/facebook-angry-emoji-algorithm/.
